# Inpatient Buprenorphine Induction for Opioid Use Disorder in Pregnancy

**DOI:** 10.7759/cureus.36376

**Published:** 2023-03-19

**Authors:** Amin Tavakoli, Kelly Donovan, Heather Sweeney, Kristen Uquillas, Brian Gordon

**Affiliations:** 1 Obstetrics and Gynecology, Los Angeles County University of Southern California Medical Center, Los Angeles, USA

**Keywords:** opioid use disorder (oud), neonatal opioid withdrawal syndrome, opioid withdrawal, medication for opioid use disorder (moud), buprenorphine

## Abstract

Objective

Buprenorphine is a commonly used medication to manage opioid use disorder, however there is limited data to guide induction protocols specifically during pregnancy. Similar to non-pregnant patients the Clinical Opiate Withdrawal Scale (COWS) is often used to guide induction and titration of buprenorphine in pregnancy. The objective of this retrospective descriptive study is to assess the inpatient buprenorphine induction patterns, treatment retention, and pregnancy outcomes among obstetric patients with opioid use disorder seeking treatment.

Study design

This was a retrospective study of obstetric patients with opioid use disorder admitted for inpatient buprenorphine induction at a large academic center between May 2015 to 2020. A descriptive analysis of the cohort, induction patterns, and dose retention after discharge were evaluated in addition to obstetric and neonatal outcomes.

Results

Sixty patients were admitted for inpatient buprenorphine induction at a median gestational age of 16.7 weeks. The median COWS score on presentation was 9. The starting dose for half of the patients (30 out of 60 patients) was 8 mg of buprenorphine, while 24 patients were started at 4 mg. The median duration of hospitalization was three days (range 2-12). The median buprenorphine dose upon discharge was 10 mg (range 4-20). Only 13 of the 35 patients (37%) who desired prenatal care at our institution returned to receive routine prenatal care. Of the 12 (20%) patients who delivered at our institution, nine were live births (75%). Among the live births, the median gestational age at delivery was 37.4 weeks, birth weight 3085 grams, and only one (8%) developed neonatal abstinence syndrome.

Conclusion

When using the Clinical Opiate Withdrawal Scale to guide inpatient buprenorphine titration for pregnant patients with opioid use disorder it takes approximately three days to establish a satisfactory maintenance dose with the median dose at discharge in this population being 10 mg. The majority of patients who followed up after hospital discharge did not need dose adjustments.

## Introduction

Opioid use disorder (OUD) has increased dramatically among pregnant patients in recent years due to the ongoing opioid epidemic, with a four-fold increase in OUD in hospitalized pregnant patients between 1999 and 2014 [1]. Between 2007 and 2016, the percentage of all pregnancy-associated deaths due to opioids in the United States doubled, reaching 10% [2]. In addition to maternal risks, untreated opioid use has been shown to have significant adverse neonatal outcomes including increased risk of fetal growth restriction, placental abruption, fetal death, preterm labor, and intrauterine passage of meconium [3]. Rates of intensive care admission for neonatal opioid withdrawal syndrome (NOWS) have also increased substantially across the country [4]. The increasing burden of OUD on perinatal health has underscored the need for improvements in OUD treatment during pregnancy. Medication for opioid use disorder (MOUD) is the standard of care treatment and has been shown to decrease overdose rates [5], yet many pregnant patients with OUD fail to receive treatment [6,7].

Buprenorphine is a high-affinity partial mu opioid agonist that has been increasingly used to treat OUD in both the general population and among pregnant patients, joining methadone (a long-acting, full mu opioid agonist) which has long been the treatment of choice in pregnancy [8]. Office-based buprenorphine therapy for obstetric patients has been shown to have better neonatal outcomes than methadone treatment. Benefits of buprenorphine use include reduction in the severity of NOWS, significantly shorter durations of neonatal treatment, and lower doses of morphine administration when compared to methadone [9].

Initiating buprenorphine before or during the first trimester has been shown to improve long-term treatment retention at 12 months postpartum [10]. Buprenorphine’s mechanism of partial mu agonism can precipitate withdrawal, requiring that patients demonstrate mild to moderate symptoms of withdrawal prior to their first dose. Difficulty timing this first dose can lead to complicated or unsuccessful inductions due to both precipitated and protracted withdrawals. These complicated inductions are associated with decreased treatment retention in non-pregnant patients [11]. In pregnant patients there is limited data to guide buprenorphine induction protocols in pregnancy, and even less literature discussing efficacy of specific protocols. Differences in pharmacokinetics during pregnancy, such as the need for higher and more frequent buprenorphine doses, further highlights the need for more data regarding buprenorphine induction and efficacy specific to pregnancy [12,13].

Guidelines for inpatient buprenorphine induction with titration based on Clinical Opiate Withdrawal Scale (COWS) scores may exist at many institutions, however variations to these guidelines are often permitted at the clinical discretion of the providers. Recognizing this permitted variation by providers, as done in this study, allows for a more accurate reflection of how this complex condition is actually being treated. The purpose of this study is to assess the inpatient buprenorphine induction patterns at a large academic center, treatment retention, and pregnancy outcomes among obstetric patients with opioid use disorder seeking treatment. Of note, data from this manuscript has been previously presented as an abstract at the 2021 Society of Maternal Fetal Medicine (SMFM) conference.

## Materials and methods

Study design and cohort selection

This study followed a retrospective cohort study design. Inclusion criteria included any pregnant patients admitted for inpatient buprenorphine induction between May 2015 and 2020 at a single institution, LAC+USC Medical Center, in Los Angeles, California. This study is designed to assess buprenorphine induction patterns (i.e. time required to establish maintenance dose, average maintenance dose on discharge, and patient retention) in addition to obstetric and neonatal outcomes of those undergoing treatment. All information was obtained via chart review of electronic medical records. Study data were collected and managed using REDCap (Research Electronic Data Capture), a secure web-based software platform designed to support data capture for research studies hosted at the University of Southern California [14,15].

Exposure

The exposure of interest was buprenorphine induction in pregnant patients with OUD. Presence and severity of OUD were determined by patient-reported opioid use as well as COWS score on initial hospital presentation [16]. Although our institution did have guidelines regarding buprenorphine induction in pregnancy at the time of this study, variations to these guidelines were permitted at the clinical discretion of the providers. The patient’s history and physical included the last time the patient used opioids, the frequency of opioid use, as well as the patient’s COWS score on admission. Buprenorphine was administered sublingually (SL) with an initial dose between 2 mg and 16 mg depending on COWS score and provider discretion. After the initial dose, additional buprenorphine doses were administered as needed when the patient was exhibiting withdrawal symptoms (COWS ≥5), usually at least 12 hours after last opioid use. After day one, the daily buprenorphine dose was calculated as the sum of the doses administered on the day prior, with the patient still able to receive additional doses as needed. 

Outcomes

The primary outcome was the duration of inpatient hospitalization for buprenorphine titration. Additional outcomes were the dose and frequency of buprenorphine administration upon discharge, need for dose adjustments after discharge as well as obstetric and neonatal outcomes in patients who returned to deliver at our institution. Specifically, the obstetric and neonatal outcomes of interest were live births, birth weight, gestational age at birth, and presence of NOWS. Presence of NOWS was determined clinically via assessment of neonatal signs such as psychomotor disturbances, problems with sleeping or feeding, and excessive crying or irritability.

Covariates

No other maternal variables were analyzed except fetal gestational age at initial presentation, which was determined via patient history of known last menstrual period or known estimated date of delivery based on outside ultrasound and/or biometric ultrasound performed at LAC+USC Medical Center. Pregnancy dating was performed in accordance with the American College of Obstetrics and Gynecology (ACOG) dating guidelines [17].

Data analysis

Univariate analysis was performed to examine the characteristics of obstetric patients at our institution desiring buprenorphine pharmacotherapy. Measures of central tendency were used for analyzing time to discharge after buprenorphine titration, maintenance buprenorphine dose at discharge, and also for gestational age and weight at birth. Descriptive statistics were used for other neonatal and obstetrics outcomes. Data analysis was completed with R (R Core Team (2021), Vienna, Austria).

## Results

Study cohort

A total of 60 obstetric patients with OUD were admitted for buprenorphine induction at LAC+USC Medical Center between May 2015 and 2020. The median gestational age of patients undergoing induction was 16.7 weeks (Table 1). The median COWS score at presentation was 9 (Table 2). 

**Table 1:** Baseline Characteristics of Study Cohort

Characteristic	Value (Total of 60 patients)
Median Maternal Age in years (range)	29 (17-42)
Median Gravidity (range)	3 (1-13)
Race	Hispanic - 18 (31.0%)
	White - 16 (27.6%)
	African American - 7 (12.1%)
	Asian - 1 (1.7%)
	Other - 4 (6.9%)
	Unknown - 12 (20.7%)
Median Gestational Age in weeks (range)	16.7 (5-39.4)
Median Body Mass Index (range)	24 (18-41)
Planned Pregnancy	Yes - 2 (4%)
	No - 43 (96%)
Desired Pregnancy	Yes - 39 (81%)
	No - 9 (19%)
Reported Substances Used n(%)	Heroin - 59 (98%)
	Prescription Opioid - 1 (2%)
	Methamphetamine - 22 (37%)
	Cocaine - 0 (0%)
	Marijuana - 7 (12%)
	Alcohol - 3 (5%)
	Tobacco - 27 (45%)
Sexually Transmitted Infection	Yes - 34 (60.7%)
	No - 22 (39.3%)
Medical Comorbidities n(%)	Asthma - 5 (8.3%)
	Chronic Hypertension - 1 (1.7%)
	Gastroesophageal reflux disease - 0 (0%)
	Hypothyroidism - 0 (0%)
	Pregestational Diabetes - 0 (0%)
Psychiatric Disorder n(%)	Anxiety - 6 (10.0%)
	Bipolar Disease - 4 (6.7%)
	Depression - 5 (8.3%)
	Schizophrenia - 1 (1.7%)
Prior Opioid Use Disorder Treatment n(%)	Buprenorphine - 12 (20%)
	Methadone - 10 (16.7%)

**Table 2:** Clinical Opiate Withdrawal Scale (COWS) Scores, Buprenorphine Dosing, and Hospital Duration

Initial COWS Score median (range)	9 (2-24)
2nd COWS Score median (range)	6 (1-16)
3rd COWS Score median (range)	4 (0-16)
Number of COWS Assessments During Admission median (range)	6 (0-28)
Buprenorphine Initial Dose n(%)	8 mg - 30 (50%)
	4 mg - 24 (40%)
	2 mg - 4 (6%)
	12 mg - 1 (2%)
	16 mg - 1 (2%)
Buprenorphine Day 1 PRN Dose median (range)	1 (0-4)
Buprenorphine Day 2 Dose median (range)	8mg (2-20)
Buprenorphine Discharge Dose median (range)	10mg (4-20)
Dosing Frequency n(%)	Daily - 57 (95%)
	Twice Daily - 3 (5%)
Hospital Duration median (range)	3 days (2-12)

Buprenorphine administration

Half (30) of the patients who underwent buprenorphine induction received 8 mg of SL buprenorphine for their first dose on Day 1 (Table 2). Two patients were started on higher doses (12 and 16 mg). These two patients were previously on buprenorphine at an outside clinic, relapsed, presented in withdrawal, and their prior confirmed dose was restarted. 

Of the 30 patients given an initial dose of 8 mg buprenorphine on Day 1, 11 (37%) required additional doses of buprenorphine that day. A total of 14 (47%) patients required additional doses on Day 2 after receiving their total calculated dose from Day 1. In the 14 patients who received additional doses on Day 2, the average dose increase was 2.4 mg (30% increase from initial Day 1 dose). The average discharge dose for this group was 13 mg of buprenorphine (62.5% increase from initial Day 1 dose). 

Of the 24 patients given an initial dose of 4 mg buprenorphine on Day 1, 15 (63%) required additional doses of buprenorphine that day, and 13 (54%) required dose increases on Day 2. In the 15 patients who received additional doses on Day 2, the average increase was 1.75 mg (44% increase from initial Day 1 dose). The average discharge dose for this group was 8 mg of buprenorphine (100% increase from initial Day 1 dose). 

Of the four patients given an initial dose of 2 mg buprenorphine on Day 1, three (75%) required additional doses of buprenorphine that day, and three (75%) required dose increases on Day 2. In the three patients who received additional doses on Day 2, the average increase was 3.0 mg (150% increase from initial Day 1 dose). The average discharge dose for this group was 11.5 mg of buprenorphine (475% increase from initial Day 1 dose). 

Primary outcome: duration of hospitalization

The median duration of hospitalization for all patients who underwent buprenorphine induction was three days, with a range of two to 12 days, excluding a patient who remained admitted 25 days for preeclampsia. 

Eight patients had hospital stays of six days or longer. Of the eight, six reached stable buprenorphine doses before day six but remained admitted for other medical problems (four) or concurrent titration of benzodiazepines for alcohol or benzodiazepine withdrawal (two). Patients who were initiated on 8 mg of buprenorphine had a median length of hospital stay of three days, whereas those who were initiated on 4 mg had a median length of stay of four days. Of the four patients who were initiated on 2 mg, one required a prolonged admission of 25 days for preeclampsia, one left against medical advice after four days, and the other two had lengths of stay of four and seven days. 

Additional outcomes

Buprenorphine doses at discharge and at the time of delivery were evaluated and compared. The median dose of buprenorphine upon discharge after buprenorphine induction was 10 mg, with a range of 4 to 20 mg. Fourteen (23%) patients were discharged on a dose less than 8 mg. Most patients were on once daily dosing, but three patients were discharged on twice daily dosing. Of the 12 patients who delivered at LAC+USC Medical Center, two patients were taking a lower dose at the time of delivery and one patient switched to methadone. The two on a lower dose were decreased per that patient’s request. The patient who switched to methadone did so because she had been on it prior to trialing buprenorphine, and she felt more comfortable with methadone. No patients required an increase in their buprenorphine dose.

Live births occurred in nine of the 12 patients who delivered at LAC+USC Medical Center (75%). There were two fetal demises at 7.43 weeks and 17.6 weeks, both shortly after admission for buprenorphine induction. There was one therapeutic abortion at 7.43 weeks. The median gestational age at time of delivery in live births was 37.4 weeks, and the median birth weight was 3085 grams. Only one neonate developed NOWS. Notably, the mother of this neonate reported recent opioid use and was started on buprenorphine induction just two days before delivery. The neonate recovered well with only nonpharmacologic management.

## Discussion

This study describes maternal and neonatal outcomes of obstetric patients undergoing buprenorphine induction from 2015 to 2020. Most patients were started on 4 or 8 mg of buprenorphine, which corresponds to starting doses seen in existing data among nonpregnant patients [18]. Patients initiated on 8 mg of buprenorphine required fewer inpatient dose adjustments and had shorter hospital stays compared to patients initiated on 4 mg or less. Most of the patients starting with either 4 mg or 2 mg buprenorphine required dose adjustments, compared to a minority of those starting with 8 mg. This data corresponds to that among nonpregnant patients, in whom lower starting doses, such as 2 mg, have been associated with complicated inductions [11]. The higher starting doses in our study also corresponded clinically to the higher COWS scores at presentation, with the median score of 9 already signaling mild withdrawal and necessitating treatment. For patients presenting without yet having withdrawal symptoms or after using long-acting opioids such as methadone, smaller doses and slower inductions may be warranted [19]. 

The median dose upon discharge was 10 mg. Three-quarters of patients were discharged on doses of 8 mg or higher. Discharge dose did not correlate with initial dose. The median dose of 10 mg corresponds with buprenorphine doses cited in other studies. A scoping review of over 3,000 obstetrical patients treated with buprenorphine found a mean dose of 13.8 mg at time of delivery [20]. While stigma around substance use disorders in pregnancy and MOUD may cause patients and providers to feel pressure to remain at a lower dose, higher doses have not been shown to worsen NOWS. Randomized trials of both buprenorphine and methadone have found no relationship between dose and adverse outcomes such as total morphine needed to treat NOWS, neonatal hospital stay, duration of NOWS treatment, estimated gestational age at delivery, 5-minute APGAR, or newborn anthropometric measurements [21,22]. Rather, higher buprenorphine doses are associated with higher levels of treatment retention during pregnancy and postpartum [20,23,24]. Although initial dose did not predict discharge dose, the more clinically significant outcomes are number of dose adjustments and length of stay, as these could influence patient withdrawal, reflect more complicated inductions, and potentially contribute to patients leaving against medical advice due to the difficulty and discomfort of reaching a stable dose. 

Patients followed through delivery ultimately had positive birth outcomes, with the majority (nine) of neonates being born full term, having normal birth weights, and avoiding NOWS. The one patient whose infant did develop NOWS had recent illicit substance use, which aligns with existing data that show NOWS to be more common among patients with treatment non-adherence and/or other substance use [19].

Strengths of this study

This study adds to the literature in that it is one of the only studies to describe buprenorphine induction procedures in pregnant patients and analyze the outcome. Patients in our study ultimately were well-controlled on doses similar to those cited in observational studies of pregnant patients using buprenorphine [20]. Likewise, our data parallel those of studies on non-pregnant adults, which show higher starting doses may be superior in preventing withdrawal and facilitating treatment adherence [11,18].

Limitations

Limitations of this observational study include small sample size and high loss to follow up. While those patients with live births ultimately had good outcomes and most infants did not have NOWS, the outcomes for the majority of patients in our study are unknown. This may largely be due to high numbers of patients who are incarcerated. After initial induction at our institution, patients who are incarcerated typically follow one of two paths for the remainder of their pregnancy. Either they remain incarcerated and receive prenatal care while in custody, returning to LAC+USC Medical Center for their delivery; or they are released from custody and then obtain prenatal care at an institution close to where they reside. High rates of incarceration are seen elsewhere in the literature, with 29.3% of patients in one study having current legal involvement and 23% lost to follow up. Those lost to follow up were more likely to have legal involvement 30 days prior to cohort enrollment [23,25]. 

While a standardized protocol existed, providers often utilized higher starting doses of buprenorphine (i.e. 4 or 8 mg) as more evidence became available in non-pregnant populations. The observational data from this study suggest benefits of these higher doses, especially 8 mg, and future randomized studies could better enumerate the differences between starting doses and other induction details. Likewise, future studies should also consider home induction, as this is increasingly common for non-pregnant patients [26]. Still, protocols for inpatient induction will remain important given that many patients with OUD are also unhoused or incarcerated.

## Conclusions

The Clinical Opiate Withdrawal Scale can be used to guide inpatient buprenorphine titration for pregnant patients with OUD, which takes approximately three days to establish a satisfactory maintenance dose of 10 mg. Similar to the general population, pregnant patients initiated at a higher dose (8 mg) had shorter hospital stays and required fewer dose adjustments compared to those initiated at lower doses (2 mg or 4 mg). The majority of patients who followed up after hospital discharge, regardless of initiation dose, did not need further dose adjustments and had good outcomes.

## References

[1] Haight SC, Ko JY, Tong VT, Bohm MK, Callaghan WM (2018). Opioid use disorder documented at delivery hospitalization - United States, 1999-2014. MMWR Morb Mortal Wkly Rep.

[2] Gemmill A, Kiang MV, Alexander MJ (2019). Trends in pregnancy-associated mortality involving opioids in the United States, 2007-2016. Am J Obstet Gynecol.

[3] Center for Substance Abuse Treatment Medication-assisted treatment for opioid addiction during pregnancy. Medication-Assisted Treatment for Opioid Addiction in Opioid Treatment Programs. Treatment Improvement Protocol (TIP) Series, No. 43.

[4] Tolia VN, Patrick SW, Bennett MM (2015). Increasing incidence of the neonatal abstinence syndrome in U.S. neonatal ICUs. N Engl J Med.

[5] Schiff DM, Nielsen T, Terplan M (2018). Fatal and nonfatal overdose among pregnant and postpartum women in Massachusetts. Obstet Gynecol.

[6] Terplan M, McNamara EJ, Chisolm MS (2012). Pregnant and non-pregnant women with substance use disorders: the gap between treatment need and receipt. J Addict Dis.

[7] Martin CE, Longinaker N, Terplan M (2015). Recent trends in treatment admissions for prescription opioid abuse during pregnancy. J Subst Abuse Treat.

[8] Young JL, Martin PR (2012). Treatment of opioid dependence in the setting of pregnancy. Psychiatr Clin North Am.

[9] Jones HE, Kaltenbach K, Heil SH (2010). Neonatal abstinence syndrome after methadone or buprenorphine exposure. N Engl J Med.

[10] O'Connor AB, Uhler B, O'Brien LM, Knuppel K (2018). Predictors of treatment retention in postpartum women prescribed buprenorphine during pregnancy. J Subst Abuse Treat.

[11] Whitley SD, Sohler NL, Kunins HV, Giovanniello A, Li X, Sacajiu G, Cunningham CO (2010). Factors associated with complicated buprenorphine inductions. J Subst Abuse Treat.

[12] Bastian JR, Chen H, Zhang H (2017). Dose-adjusted plasma concentrations of sublingual buprenorphine are lower during than after pregnancy. Am J Obstet Gynecol.

[13] Caritis SN, Bastian JR, Zhang H (2017). An evidence-based recommendation to increase the dosing frequency of buprenorphine during pregnancy. Am J Obstet Gynecol.

[14] Harris PA, Taylor R, Thielke R, Payne J, Gonzalez N, Conde JG (2009). Research electronic data capture (REDCap)--a metadata-driven methodology and workflow process for providing translational research informatics support. J Biomed Inform.

[15] Harris PA, Taylor R, Minor BL (2019). The REDCap consortium: building an international community of software platform partners. J Biomed Inform.

[16] Wesson DR, Ling W (2003). The Clinical Opiate Withdrawal Scale (COWS). J Psychoactive Drugs.

[17] (2017). Committee Opinion No 700: methods for estimating the due date. Obstet Gynecol.

[18] Cao SS, Dunham SI, Simpson SA (2020). Prescribing buprenorphine for opioid use disorders in the ED: a review of best practices, barriers, and future directions. Open Access Emerg Med.

[19] Johnson S, Martin PR (2018). Transitioning from methadone to buprenorphine maintenance in management of opioid use disorder during pregnancy. Am J Drug Alcohol Abuse.

[20] Martin CE, Shadowen C, Thakkar B, Oakes T, Gal TS, Moeller FG (2020). Buprenorphine dosing for the treatment of opioid use disorder through pregnancy and postpartum. Curr Treat Options Psychiatry.

[21] Jones HE, Jansson LM, O'Grady KE, Kaltenbach K (2013). The relationship between maternal methadone dose at delivery and neonatal outcome: methodological and design considerations. Neurotoxicol Teratol.

[22] Jones HE, Dengler E, Garrison A (2014). Neonatal outcomes and their relationship to maternal buprenorphine dose during pregnancy. Drug Alcohol Depend.

[23] Ray-Griffith S, Tharp E, Coker JL, Catlin D, Knight B, Stowe ZN (2021). Buprenorphine medication for opioid use disorder: a study of factors associated with postpartum treatment retention. Am J Addict.

[24] Coker JL, Catlin D, Ray-Griffith S, Knight B, Stowe ZN (2018). Buprenorphine medication-assisted treatment during pregnancy: an exploratory factor analysis associated with adherence. Drug Alcohol Depend.

[25] Brogly SB, Saia KE, Werler MM, Regan E, Hernández-Díaz S (2018). Prenatal treatment and outcomes of women with opioid use disorder. Obstet Gynecol.

[26] (2018). Clinical Guidance for Treating Pregnant and Parenting Women With Opioid Use Disorder and Their Infants. https://store.samhsa.gov/product/Clinical-Guidance-for-Treating-Pregnant-and-Parenting-Women-With-Opioid-Use-Disorder-and-Their-Infants/SMA18-5054.

